# CT Appearance of Hepatocellular Carcinoma after Locoregional Treatments: A Comprehensive Review

**DOI:** 10.1155/2015/670965

**Published:** 2015-12-22

**Authors:** Daniel Marin, Salvatore Cappabianca, Nicola Serra, Assunta Sica, Francesco Lassandro, Roberto D'Angelo, Michelearcangelo La Porta, Francesco Fiore, Francesco Somma

**Affiliations:** ^1^Department of Radiology, Duke University, Durham, NC 27701, USA; ^2^Department of Radiology, Second University of Naples, 80131 Naples, Italy; ^3^Department of Radiology, Monaldi Hospital, 80131 Naples, Italy; ^4^Department of Interventional Radiology, National Cancer Institute, “Fond. Pascale”, 80131 Naples, Italy; ^5^Department of Radiology, UOC San Severo Hospital, 71016 San Severo, Italy

## Abstract

Hepatocellular carcinoma (HCC) is a major health problem worldwide, affecting more than 600,000 new patients per year. Curative treatments are available in a small percentage of patients, while most of them present in stages requiring locoregional treatments such as thermoablation, transarterial chemoembolization, and/or radioembolization. These therapies result in specific imaging features that the general radiologist has to be aware of in order to assess the response to treatment and to correctly manage the follow-up of treated patients. Multiphasic helical computed tomography has become a popular imaging modality for detecting hypervascular tumors and characterizing liver lesions. On this basis, many staging and diagnostic systems have been proposed for evaluating response to all different existing strategies. Radiofrequencies and microwaves generate thermoablation of tumors, and transarterial chemoembolization exploits the double effect of the locoregional administration of drugs and embolizing particles. Eventually radioembolization uses a beta-emitting isotope to induce necrosis. Therefore, the aim of this comprehensive review is to analyze and compare CT imaging appearance of HCC after various locoregional treatments, with regard to specific indications for all possible procedures.

## 1. Introduction

Hepatocellular carcinoma (HCC) is a major health problem worldwide, affecting more than 600,000 new patients per year [1]. Curative treatments are hepatic resection, liver transplantation, and percutaneous ablation [2]. Unfortunately, such treatments are generally indicated in less than 20% of patients [3, 4], while most of them present with advanced-stage disease or multifocal tumor, contraindicating any radical treatment option [5]. To date, several alternative approaches have been proposed, both systemic or locoregional [6]. Some of them such as transarterial chemoembolization (TACE) or radioembolization (TARE) are also used as a bridge to liver transplantation or to downstage tumors exceeding Milan criteria [7–11]. Some others, such as thermoablation using microwaves or radiofrequency, are designed to destroy tumors by heating tissue to temperatures higher than 60°C [12–14]. Irrespective of which locoregional treatment is performed, imaging plays a pivotal role in the follow-up of hepatic tumors, as it is the means by which local treatment efficacy, recurrent disease, and therapy-induced complications are evaluated [15]. Nowadays, multidetector computed tomography (MDCT) is still the most widely used imaging technique to describe the appearance of hepatic tumors treated with locoregional therapies. Moreover, it allows us to accurately assess the response to therapy through the evaluation of tumor size, tumor margins, tumor necrosis, and early detection of residual or recurrent tumor and new tumor. The evaluation of treatment success is crucial in the next treatment decisions and for prognosis [16]. Therefore, the aim of this paper is to review and compare CT imaging appearance of HCC after various locoregional treatments.

## 2. Locoregional Treatment Options for HCC

**Figure 1:**
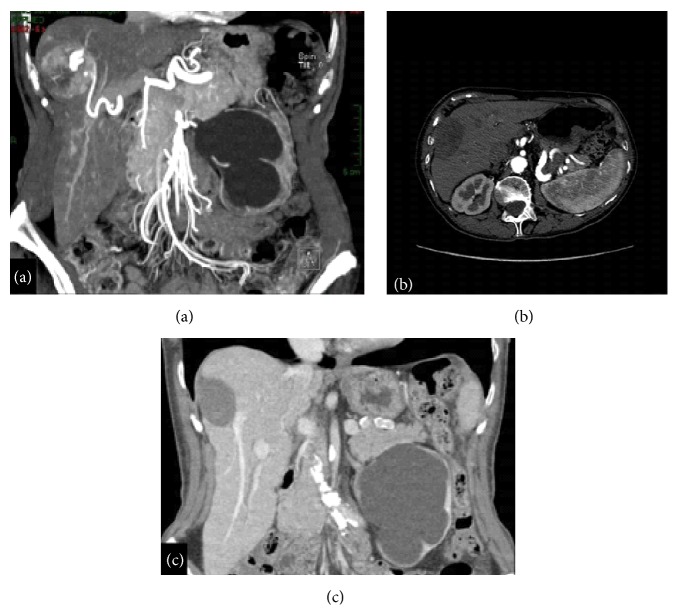
RFA. HCC of the 7th segment treated with RFA: (a) before treatment, MDCT arterial phase with multiplanar reconstruction; (b) 6 months after treatment, MDCT arterial phase; (c) 12 months after treatment, MDCT portal phase with multiplanar reconstruction.

The Barcelona Clinic Liver Cancer classification has been widely accepted as guideline for all therapies available in different stages of HCC [17]. This staging system links the stage of the disease to a specific treatment strategy, such as curative treatments or palliative therapies. Very early-stage (BCLC Stage 0) and early-stage HCC (BCLC Stage A) are still amenable to potentially curative therapies, such as hepatic resection, liver transplantation [2], providing best 5-year survival of more than 50% [18]. In case of focused disease with no extrahepatic spread, resection is the first-choice treatment, even if transplantation is preferred by many authors because, if possible, it removes underlying diseased liver that predisposes to the development of new hepatic lesions [19]. However, most patients show intermediate (BCLC Stage B) or advanced HCC (BCLC Stage C) at presentation, thus making sorafenib or locoregional treatments recommended [20]. These therapies have the advantages of preserving a larger part of hepatic parenchyma with overall less morbidity and mortality compared with resection, thanks to reduced intraoperative blood loss [21–24]. The most used locoregional treatment is the imaging-guided percutaneous thermal ablation using radiofrequency (RFA) or microwaves, transarterial chemoembolization (TACE), and radioembolization (TARE). Moreover, RFA is also used during nonconventional liver resection to obtain parenchymal dissection by creating a zone of coagulative necrosis along the transection plane [25]. This technique is indicated in patients with preserved liver function and single HCC, ideally in subcapsular position [26], reducing the risk of intraoperative blood loss when compared with conventional liver resection [25, 27]. Instead, percutaneous RFA is indicated for early-stage HCC in patients who are not suitable candidates for resection. In particular, this technique showed being more efficient than percutaneous ethanol ablation in tumors with a diameter greater than 3 cm [28]. Differently from ethanol, thermal ablation is not chemical but uses high temperatures to induce cellular disruption and tissue coagulation necrosis [29]. Patients with very early-stage HCC show complete response rates of 97% with 5-year survival rates of 68% [30]. However, vessels greater than 3 mm in diameter surrounding the site of ablation may limit RFA action, due to heat loss caused by perfusion-mediated tissue cooling [10]. This is not a limit for methods using microwaves. Indeed, despite the little amount of studies on effectiveness, microwave methods are currently emerging thanks to the many advantages, such as larger tumor ablation volumes, faster tumor ablation, and the resistance to tissue cooling due to adjacent vessels [31]. Intermediate-stage and advanced-stage HCC with no extrahepatic spread are rather suitable for transarterial therapies, such as TACE or TARE. Contrary to normal liver, HCC receives blood supply almost entirely by hepatic artery, thus making transarterial therapies effective on HCC lesions only. Iodized oil acts as a drug carrier, while embolizing particles occlude the tumor feeding arteries. On the basis of randomized controlled trials [32–34], TACE has been recommended by BCLC as the standard of choice in case of multiple or big lesions with no vascular invasion or extrahepatic spread and for lesions not accessible percutaneously. This method consists of a transarterial administration of chemotherapy, mostly Doxorubicin, mixed with iodized oil (Lipiodol, Guerbet, France), followed by the superselective injection of embolizing particles (polyvinyl alcohol (PVA)) [35]. On the other hand, TARE has emerged for the treatment of advanced-stage HCCs, that is, in patients nonresponding to TACE, in elderly patients with large HCCs, in case of vascular invasion, and prior to liver resection in order to downstage tumor [36] (Figure 1). It consists of releasing microspheres containing yttrium-90, a *β*-emitting isotope, straight in the tumor feeding arteries after superselective catheterization. In this way, high-energy, low-penetration radiation causes tumor destruction by coagulative necrosis and avascularity [37]. Some studies have shown the efficacy of this therapy to be similar to TACE, with lower toxicity [38–40]. TARE has also shown an overall survival outcome similar to sorafenib, in particular in patients with segmental and main portal vein tumor thrombosis [41, 42].

## 3. MDCT Technique for HCC Evaluation

**Figure 2:**
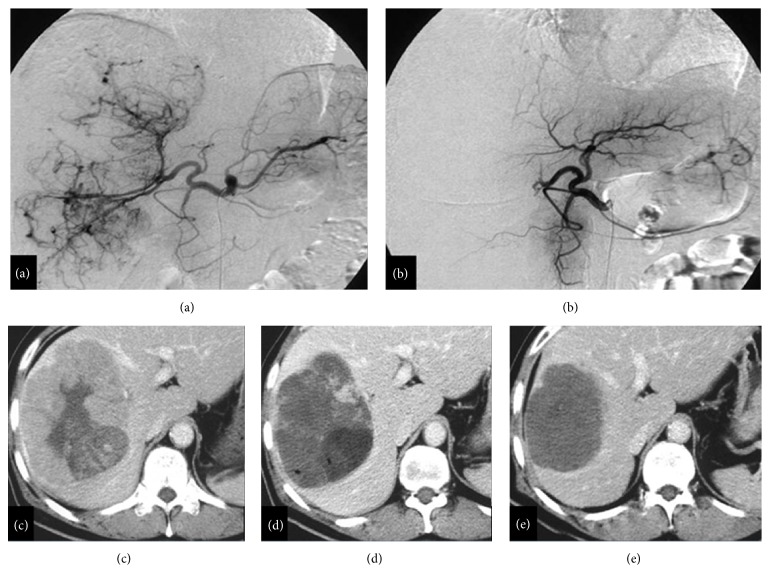
TACE. Large HCC treated with TACE: (a) before treatment, angiography; (b) after treatment, angiography; (c) before treatment, MDCT arterial phase; (d) 1-month assessment control after treatment, MDCT arterial phase; (e) 12 months after treatment, MDCT arterial phase.

Multiphasic helical CT has become a popular imaging modality for detecting hypervascular tumors and characterizing liver lesions. In patients with cirrhosis, MDCT performed during the hepatic arterial phase and portal venous phase is often used as the first-line diagnostic modality for detection of HCC, follow-up after local treatment or surgical excision, and assessment of hemodynamic changes in the liver [43]. Despite its high reliability in examining patients with HCC, it is unclear whether biphasic MDCT is the best technique to evaluate the effects of locoregional therapies and the possibility of tumor recurrence. Contrast-enhanced magnetic resonance imaging (MRI) has also been assessed as a valuable method to study patients with HCC, especially after transcatheter arterial therapies, such as TACE and TARE [44]. In particular, lesions treated with RFA or TACE typically undergo coagulative hemorrhagic necrosis that may appear hyperintense on unenhanced T1-weighted imaging, making contrast-enhanced evaluation difficult [45]. Image subtraction techniques with MRI have been shown to be beneficial in depicting residual enhancement, with excellent correlation with histopathologic degree of tumor necrosis [46]. However, the increased cost and comparative lack of availability of this modality make MDCT the mainstay of liver and HCC imaging for both initial tumor characterization and posttreatment follow-up for response assessment [47–49]. MDCT uses 16, 62, 128, or even more contiguous detectors to increase effective pitch without consequent loss of spatial resolution along the axis of scanning, thus allowing thin-section images to be obtained in a single breath-hold with greatly improved speed and longitudinal resolution, resulting in high-resolution multiplanar reformations. For patients with HCC eligible for liver transplantation, the United Network for Organ Sharing currently recommends the use of a quadruple-phase CT protocol that includes unenhanced images (to characterize residual enhancement in posttreatment cases), a single late arterial phase based on a bolus-tracking method (for accurate peak arterial enhancement), a portal venous phase, and a late venous phase, respectively, at 70 and 120 seconds after iodine contrast injection at a rate of 4-5 mL/s [50] (Figure 2).

## 4. Assessment of Tumor Response in HCC

In the past, tumor response evaluation systems have focused on anatomic biomarkers. The Response Evaluation Criteria in Solid Tumors (RECIST) considered the largest diameter of the lesion and was intended to evaluate changes in tumor size over months to years after systemic treatments, without taking into account changes in tumor tissue composition [51, 52]. Similarly, the World Health Organization (WHO) guidelines consider bidimensional perpendicular measurements [53]. However, these systems fail in evaluating the outcome of locoregional therapies, because the aim of these treatments is to obtain the tumor necrosis rather than the lesion removal. Indeed, after these therapies, HCC is likely to increase in size because of intratumoral edema, hemorrhage, or necrosis [54]. Due to these limitations, new criteria have been proposed by European Association for the Study of the Liver (EASL) [26], which modified the previous bidimensional measurements proposed by the WHO guidelines. More recently, the modified RECIST has been introduced in order to address many shortcomings affecting older evaluation systems [55]. As the unmodified RECIST, the modified version uses the single largest diameter of the tumor, considering only the component enhancing during the arterial phase [52]. This system is based on dynamic MDCT examination performed 1 month after locoregional therapy and has been endorsed by EASL and European Organization for Research and Treatment of Cancer (EORTC) [56].

However, even modified RECIST has some limitations, especially in the assessment of response after RFA and TARE, since these criteria are difficult to apply with confidence in the measurement of diffusely necrotic lesions with interspersed viable components [57]. Therefore, a previous study proposed a reduction in volume as standard of reference for tumor response [51], with partial response representing a volume reduction of 65% according to standard oncologic criteria.

## 5. Appearance of Treated HCC

### 5.1. Overall Considerations

Due to the exceedingly complex therapeutic approach to HCC, a therapy-tailored imaging evaluation of tumor response in HCC is mandatory [58]. Indeed, the correct evaluation of posttherapeutic changes in tumor viability and vascularization may alter the management of the patient, with regard to the treatments to perform. This is particularly true in case of locoregional treatments, whose ultimate goal is the tumor cell death and necrosis, with sparing of healthy surrounding tissue [59].

### 5.2. Imaging after RFA

The aim of this kind of treatment is to generate an area of thermocoagulation larger than the tumor, by forming a necrotic scar that usually shrinks very slowly with time. This fact makes WHO criteria not applicable in the response assessment of thermal ablation [60]. Previous reports [14, 61–63] show the reduced value of unenhanced US in the evaluation of RFA efficacy, due to the similar appearance of necrotic and viable tumor tissue on US images. The use of contrast medium may help [64]. Anyway, contrast-enhanced CT or MR imaging is at present considered the most useful modalities, using as major criterion of efficacy the absence of enhancement in RF-induced necrosis. Moreover some reports suggested high confidence of these modalities in the identification of the ablation area, with only 2 mm miscalculation of the coagulated necrosis measured at histologic examination [65, 66]. On unenhanced CT images, areas treated with RFA generally appear as homogeneously hypoattenuating or heterogeneous with interspersed hyperattenuating foci in a hypoattenuating area. Contrast-enhanced CT images may show no enhancement in case of successful treatment or some area of irregular enhancement in case of incomplete ablation. In the latter case, the enhanced area may appear as a thin rim surrounding the treated lesion or as a thick nodule abutting the site of RF ablation. Local regrowths are usually seen as irregular thickening of one margin of the treated area. Peripheral recurrence may be explained by lower energy deposition and reduced heating in the locations further from the needle electrode. Moreover, tissue perfusion lowers heat accumulation by cooling, thus allowing more likely recurrence close to larger vessels abutting the site of ablation [65]. Peripheral thin and regular rim of enhancement (<1 mm) may be seen at the later phase after contrast medium administration and represents a ring of vascularized inflammatory reaction with granulation tissue surrounding necrosis [67]. This finding should never be diagnosed as regrowth, whose contrast enhancement is always thicker and irregular [62]. Wedge-shaped enhancement in the liver parenchyma adjacent to the ablation site has also been described [15] and is probably due to peripheral arterioportal shunts caused either mechanically by needle puncture or physically by thermal damage. Treatment-related complications to look for during post-RFA imaging are intrahepatic abscesses at the site of ablation, necrosis along the path of the RF electrode, and segmental dilation of intrahepatic bile ducts in contact with the ablation area.

### 5.3. Imaging after Microwaves

Percutaneous microwave coagulation therapy is considered to be a possible treatment of unresectable small HCCs [68, 69], due to definite tissue necrosis around the electrode and hemostatic effect of microwave irradiation [70]. Contrast-enhanced CT is commonly used to assess the complete necrosis of the tissue and possible recurrence [71, 72]. However, CT findings after HCC ablation using microwaves may be challenging and sometimes tricky. Indeed, the use of this percutaneous modality often causes an early enhancement of the normal hepatic tissue around the treated area. This postprocedural sign is likely to be a transient reaction of normal tissue to thermal damage, as it is detectable also in other procedures inducing tissue heating. On histologic specimen, this finding has been explained with a massive sinusoidal dilatation at the boundary between the coagulated area and the surrounding normal tissue, determining a peripheral granulation tissue and fibrosis after treatment [73, 74]. After treatment, an increase of arterial blood flow may occur at the margin of the treated area, leading to hepatic hypoperfusion during the arterial phase as a result of inflammation changes caused by microwaves irradiation as well as radiofrequency thermal ablation [67, 75]. Moreover, the formation of arterioportal shunts is another source of abnormal enhancement mimicking hypervascular lesions [76, 77]. The arterioportal shunts are caused by the piercing of an artery in the portal tract by the needle. Therefore, they may be recognized as wedge-shaped areas of enhancement during the arterial phase on CT [78–80] and are essentially due to the number of punctures performed rather than to thermal changes.

### 5.4. Imaging after TACE

This modality consists of transarterial administration of a mixture of chemotherapy and embolizing particles directly in the tumor feeding arteries, after a superselective catheterization. Contrary to normal liver, HCC receives blood supply almost entirely from the hepatic artery, and this fact allows drug accumulation preferentially into HCC lesions. CT images evaluation of tumor response to TACE is based on the assumption that the necrotic area of the tumor retains iodized oil, with enhanced foci representing viable tissue. However, beam hardening artifacts due to iodized oil retention may conceal arterial enhancement [57]. Therefore, the use of unenhanced phase is crucial to detect any additional foci of viable tumor, when compared to biphasic CT [81]. In this case, an HCC treated with TACE is to be considered as viable if showing hyperattenuation or isoattenuation on hepatic arterial phase and hypoattenuation on unenhanced and portal venous phases. A thin peripheral pseudocapsule enhanced on hepatic arterial and delayed phases may be visualized, such as other arterioportal shunts due to small hepatic arteries chemically injured iodized oil. All of these lesions differ from viable tumor for the absence of any sign of washout. Possible complications of this therapy are hepatic artery dissection or thrombosis, biloma, hepatic abscess, and embolization of nontarget vessels, which may cause gastrointestinal ulcers, skin ulcerations, and/or cholecystitis [82, 83]. Moreover, this therapy often results in a postembolization syndrome that occurs in 60–80% of patients and consists of fatigue, transient abdominal pain, ileus, fever, and increased serum levels of liver enzymes and bilirubin [84]. Different procedures have been proposed to avoid this syndrome, such as the use of drug eluting beads [85], or the replacement of chemotherapy with ethanol [86], whose imaging does not differ from the conventional TACE.

### 5.5. Imaging after TARE

**Figure 3:**
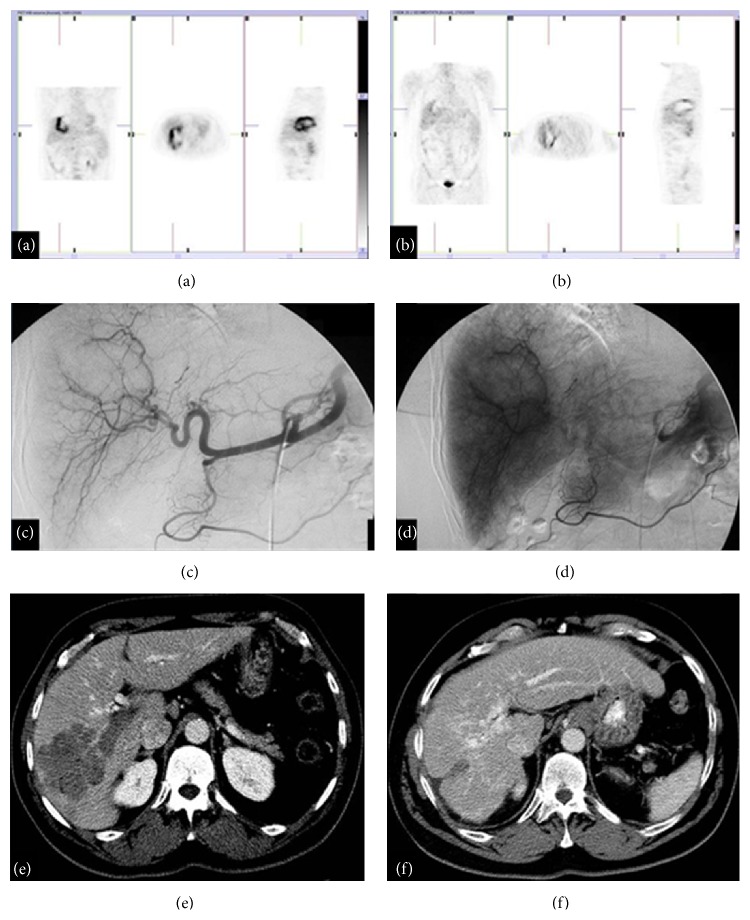
TARE. HCC treated with TARE: (a) before treatment, PET; (b) after treatment, PET; (c)-(d) during the procedure, angiography; (e) before treatment, MDCT arterial phase; (f) 12 months after treatment, MDCT arterial phase.

Radioembolization is an emerging transarterial therapy for the treatment of hepatic malignancies, involving the administration of micron-sized radioactive particles featuring yttrium 90 (^90^Y), a pure beta emitter [87] (Figure 3). Once these particles lodge in the tumor feeding arterioles, they impart a very intense local radiotherapeutic effect [88], penetrating the surrounding tissue for approximately 1 cm in diameter. Before decaying to inactive zirconium 90, the emitting particles allow the administration of up to 150 Gy to specific target areas of the liver [89]. The carrier is a microsphere ranging from 20 to 60 *μ*m in diameter, with the radioactive element bound directly in the resin (SIR spheres) or an integral constituent of the glass (TheraSphere). The predominance of arterial blood supply to the tumor grants a preferential deposition of microspheres in the lesions, minimizing irradiation to the normal parenchyma [90]. As the other ablative therapies, TARE induces an area of coagulative necrosis and relative avascularity with an overall reduction in tumor size, as a result of the lethal insult to cancer cells [26, 91]. Follow-up imaging is usually performed with multiphase CT 30 days after treatment and at regular 3-month intervals thereafter. On unenhanced CT images, coagulative necrosis generally results in homogeneously hypoattenuating area. Although uncommon, complete disappearance of tumor with no enhancement of the treated lesion may occasionally be seen. Differently from complete response, a partial response is seen in case of viable tumor volume reduction of more than 65% [51]. Other posttreatment findings are peritumoral edema and hemorrhage, due to a sort of inflammatory reaction to the intense radiation effects of ^90^Y. This sign is tricky, when associated with apparent lesion enlargement and tumor progression if the assessment is made on the basis of the sole lesion size [92]. Another possible pitfall is the ring enhancement, due to the preferential flow of blood vessels to the periphery of the tumors as well as the intense radiation effect. Previous studies have shown that after TARE this finding represents fibrous rather than residual viable tissue [93] and may persist for months without necessarily implying residual tumor [94]. Contralateral liver hypertrophy has also been demonstrated in patients receiving TARE, with no alteration of normal liver function [95]. Further findings after TARE are capsular retraction, hepatic fibrosis, and portal hypertension, probably due to shrinkage of the tumor with resultant scar formation and nodularity in uninvolved area [92]. Eventually, in case of lesions close to the Glisson capsule and the right pleural space, the induced radiation may cause reactive perihepatic fluid and pleural effusions [96]. Hepatic abscess, biliary dyskinesia and cholecystitis, biloma and biliary necrosis, and radiation hepatitis may all represent complications of this therapy. Also peptic ulceration and gastritis are known complications of radioactive ^90^Y microsphere treatment, when deposited outside of the desired location [97].

## 6. Conclusion

The recent progress in HCC treatment involves the development of several locoregional therapies that allow a focused aggression on hepatic lesions, while sparing the surrounding normal parenchyma. The posttreatment evaluation of tumor response is a crucial milestone in directing the patient management, thus making the imaging appearance of treated HCC essential for accurately assessing treatment response. Therefore, the HCC appearance on multiphase CT after locoregional therapies is a challenging matter for every radiologist, who is asked to be able to distinguish the normal posttreatment alterations from residual or recurrent disease.
